# Prevalence of target organ damage in hypertensive subjects attending primary care: C.V.P.C. study (epidemiological cardio-vascular study in primary care)

**DOI:** 10.1186/1471-2296-12-75

**Published:** 2011-07-14

**Authors:** Athanasia Papazafiropoulou, Efstathios Skliros, Alexios Sotiropoulos, Christos Papafragos, Aristofanis Gikas,4, Ourania Apostolou, Hariklia Kaliora, Charalambos Tountas

**Affiliations:** 1 Hellenic Association of Research and Continuing Education in Primary Care, Athens, Greece; 2 3rd Department of Internal Medicine and Center of Diabetes, General Hospital of Nikaia "Ag. Panteleimon" - Piraeus, Greece; 3 Nemea Health Center, Nemea, Korinthia, Greece; 4 Department of General Practice, Health Centre of Kalivia, Kalivia-Lagonisi, Athens, Greece

**Keywords:** Primary care, hypertension, target organ damage, left ventricular hypertrophy

## Abstract

**Background:**

Except for the established risk factors, presence of target organ damage has an important role in the treatment of hypertensive subjects. The aim of the present study was to estimate the prevalence of target organ damage in primary care subjects.

**Methods:**

This multi-centre, cross-sectional survey of 115 primary care physicians recruited 1095 consecutive subjects with hypertension: 611 men (55.8%); and 484 women (44.2%). A detailed history for the presence of cardiovascular disease and a thorough clinical examination was performed to each subject.

**Results:**

Of the total study population, 44.5% (n = 487) had target organ damage (33.0% had left ventricular hypertrophy, 21.8% increased carotid intima media thickness, 11.0% elevated plasma creatinine levels and 14.6% microalbuminuria). Target organ damage was more prevalent in males than in females (P = 0.05). In addition, males had more often increased carotid intima media thickness than females (P = 0.009). On the contrary, females had more often microalbuminuria (P = 0.06) than males. No differences were observed between the two genders regarding left ventricular hypertrophy (P = 0.35) and elevated plasma creatinine levels (P = 0.21). Logistic regression analysis showed associations between target organ damage and dyslipidemia (P < 0.001), presence of metabolic syndrome (P = 0.005), diabetes (P < 0.001) and coronary artery disease (P < 0.001).

**Conclusion:**

A significant proportion of hypertensive subjects in primary care had documented associated target organ damage, with left ventricular hypertrophy being the most prevalent target organ damage.

## Background

Arterial hypertension has been recognized as one of the major cardiovascular risk factors and management of hypertensive subjects still remains a challenge for the clinicians [1]. Except for the established risk factors [age, gender, family history of cardiovascular disease, smoking, obesity, dyslipidemia and diabetes mellitus (DM)], presence of subclinical target organ damage (TOD) (left ventricular hypertrophy, carotid atheromatosis and renal impairment) has an important role in the treatment of hypertensive subjects and the prevention of cardiovascular disease [2,3].

It has been showed that subclinical TOD is a marker of increased cardiovascular morbidity and mortality [4-9]. Hypertensive subjects with left ventricular hypertrophy (LVH) [4], increased carotid intima media thickness (IMT) [5] and microalbuminuria [6-9] show increased cardiovascular risk. In addition, hypertensive subjects with apparent macrovascular disease [cerebrovascular disease, coronary artery disease (CAD) and peripheral artery disease] have increased cardiovascular risk [9]. Therefore, knowledge of the presence of TOD is of major importance for the optimal management of subjects with arterial hypertension. However, little data exist regarding the prevalence of TOD in primary care subjects in our country. The aim of the present study was to estimate the prevalence of TOD in primary care subjects.

## Methods

### Population

The C.V.P.C. study (Epidemiological Cardio-Vascular Study in Primary Care) was a multi-center, cross-sectional survey which was carried out in order to determine the prevalence of TOD in subjects with hypertension in primary care. The survey included 1095 subjects with a known history of hypertension, 611 men (55.8%) and 484 women (44.2%) who consecutively attended 115 Primary care physicians from 1-10-2009 until 31-10-2009. Only 21 subjects refused to take part in the study. Patient's medical history recording and physical examination was performed by the physicians that took part in the study. All data were documented in patients' records.

A detailed history for the presence of cardiovascular disease, TOD, current medication, information about other diseases and smoking habits was obtained, and a physical examination was performed. Body weight with subjects in light clothing without shoes and height was measured and body mass index (BMI) was calculated. Waist circumference was measured with a soft tape on standing, midway between the lowest rib and the iliac crest. The biochemical parameters were recorded from laboratory testing in the three months prior to consultation (or in the days after consultation if no such prior testing proved available) at local biochemical laboratories.

Blood pressure was measured three consecutive times, one minute in apart, in the sitting position after 5 minutes rest period using an appropriate cuff size. All the measurements performed using the A & D UA-705 Upper Arm Blood Pressure Monitor The mean values of the last 2 measurements was calculated and used in the analysis. Arterial hypertension was defined according to the current guidelines [10], when systolic was ≥ 140 mmHg or and/or diastolic blood pressure was ≥ 90 mmHg or when the patients were on antihypertensive treatment. In addition the type of the antihypertensive treatment was recorded. The controlled hypertension definition was based on systolic blood pressure ≤ 140 mmHg and diastolic blood pressure ≤ 85 mmHg in subjects taking antihypertensive medications.

Dyslipidemia was defined when patients were on statin treatment. CAD was defined as presence of angina, history of previous myocardial infarction, positive stress testing, revascularization procedures or stenosis > 50% at the coronary arteries. DM was self-reported and defined as current use of antidiabetic treatment. Renal impairment was defined as plasma creatinine levels ≥ 1.3 mg/dl. Microalbuminuria was defined as 24 hour urinary albumin of 30-300 mg/dl. Presence of LVH and increased carotid IMT were reported according to subject's recent ultrasound examination. Subjects having three or more of the following criteria (according to the NCEP ATP III report) [11], were defined as having the metabolic syndrome (MS): abdominal obesity (waist circumference > 102 cm in men and > 88 cm in women), triglycerides ≥ 150 mg/dl, HDL cholesterol: ≤ 40 mg/dl in men and ≤ 50 mg/dl in women, high blood pressure: ≥ 130/85 mmHg or use of antihypertensive drugs and high fasting plasma glucose: ≥ 100 mg/dl or treatment for diabetes mellitus. Measurement of carotid IMT was performed by carotid ultrasound examination that measured the thickness of the intimal and medial layers of the arterial wall (IMT >0.9 mm in the common carotid artery is a known cardiovascular risk factor) [12]. Retinal examination was carried out by experienced ophthalmologists with no prior knowledge of the participants' blood pressure level.

The study protocol was reviewed by the bioethical committee of the Hellenic Association of Research and Continuing Education in Primary Care and a written consent was obtained from all participants.

### Statistical analyses

Data are expressed as mean ± standard deviation (SD). Student's *t*-tests and pearson's χ^2 ^test was used to compare between-groups differences. Logistic regression analysis with a backward stepwise approach was employed to identify variables associated with TOD. Relative risks (RR) were calculated from logistic regression models. Any P value ≤ 0.05 (two-tailed) was considered statistically significant. Data were analyzed using SPSS v 15.0 (SPSS, Chicago, IL, USA).

## Results

The basic demographic characteristics of the study subjects are showed in Table 1. Two hundred eighty one (25.7%) subjects had a family history of cardiovascular disease and 28.4% (n = 311) patients had MS. Smokers were 38.9% of the study population. The most frequent co-morbidities were DM (25.8%) and dyslipidemia (59.5%); followed by peripheral artery disease (12.2%), CAD (11.9%), retinopathy (7.2%), heart failure (7.0%) and cerebrovascular disease (4.9%).

**Table 1:** Characteristics of the studied population [data are showed as Mean ± SD or *n *(%)]

	*Men*	*Women*	*All*	P*
Mean age (years)	61.7 ± 10.1	62.1 ± 10.8	61.9 ± 10.5	0.46
Systolic blood pressure (mmHg)	137.3 ± 18.6	134.2 ± 14.7	138.7 ± 17.4	0.54
Diastolic blood pressure (mmHg)	84.5 ± 11.2	80.6 ± 8.7	83.3 ± 10.3	0.62
Body mass index (kg/m^2^)	29.0 ± 8.2	28.8 ± 13.2	28.9 ± 10.6	0.71
Waist circumference (cm)	104.3 ± 10.3	102.5 ± 10.0	104.0 ± 10.2	0.49
Smoking status	305 (50.6)	121 (25.6)	426 (38.9)	< 0.001
Diabetes mellitus	160 (26.6)	123 (26.0)	283 (25.8)	0.81
Metabolic syndrome	174 (29.1)	137 (29.2)	311 (28.4)	0.96
Heart failure	40 (6.6)	37 (7.8)	77 (7.0)	0.46
Coronary artery disease	102 (16.9)	28 (5.9)	130 (11.9)	< 0.001
Dyslipidemia	366 (60.4)	285 (59.6)	651 (59.5)	0.97
Stroke	29 (4.8)	25 (5.2)	54 (4.9)	0.06
Peripheral artery disease	76 (12.6)	58 (12.3)	134 (12.2)	0.89

P-values refer to the comparisons between two genders.

### Blood pressure control

56.8% of the subjects, (n = 622) had blood pressure levels that were considered well-controlled (< 140/85 mmHg). The above targets for blood pressure were achieved by 63.1% of the subjects with CAD, 53.5% of the subjects with TOD and 46.6% of the diabetic subjects. Of the subjects achieved blood pressure levels 12.5% were on single anti-hypertensive agent, 60.0% on dual anti-hypertensive agents and 27.5% were prescribed ≥3 anti-hypertensive drugs.

### Target organ damage

Of the total study population, 44.5% (n = 487) had documented TOD (33.0% had LVH, 21.8% increased carotid IMT, 11.0% elevated plasma creatinine levels and 14.6% microalbuminuria). TOD was more prevalent in males than in females (49.1% vs. 43.0%, respectively, P = 0.05). In addition, males had more often increased carotid IMT than females (25.5% vs. 18.8%, respectively, P = 0.009). On the contrary, females had more often microalbuminuria (17.5% vs. 13.4%, respectively, P = 0.06) than males. No differences were observed between the two genders regarding LVH (34.5% vs. 31.9%, respectively, P = 0.35) and elevated plasma creatinine levels (10.3% vs. 12.9%, respectively, P = 0.21) (Table 2).

**Table 2:** Prevalence of target organ damage among hypertensive subjects attending primary care

*Target organ damage*	*Men*	*Women*	*All*	P*
Left ventricular hypertrophy	207 (34.5)	154 (31.9)	361 (33.0)	0.35
Increased carotid intima media thickness	152 (25.5)	87 (18.8)	239 (21.8)	0.009
Elevated plasma creatinine levels	61 (10.3)	59 (12.9)	120 (11.0)	0.22
Microalbuminuria	79 (13.4)	81 (17.5)	160 (14.6)	0.06

P-values refer to the comparisons between two genders.

Of the subjects with dyslipidemia, 53.5% (n = 348) had documented TOD (41.0% had LVH, 27.5% increased carotid IMT, 14.3% elevated plasma creatinine levels and 18.6% microalbuminuria). Of the subjects with MS, 61.7% (n = 192) had documented TOD (49.2% had LVH, 29.9% increased carotid IMT, 21.5% elevated plasma creatinine levels and 30.9% microalbuminuria). Of the diabetic subjects, 62.2% (n = 176) had documented TOD (45.9% had LVH, 33.2% increased carotid IMT, 27.2% elevated plasma creatinine levels and 36.0% microalbuminuria).

Logistic regression analysis showed associations between TOD and dyslipidemia [odds ratio (OR): 2.04, 95% Confidence Intervals (95% CI): 1.52-2.73, P < 0.001], presence of MS (OR = 1.64, 95% CI: 1.16-2.31, P = 0.005), DM (OR = 1.93, 95% CI: 1.36-2.7, P < 0.001) and CAD (OR = 4.06, 95% CI: 2.44-6.76, P < 0.001).

## Discussion

In the present study we showed that a significant proportion of hypertensive subjects in primary care had documented associated TOD. LVH was the most prevalent TOD followed by increased carotid IMT and renal impairment. In addition, TOD was more prevalent in hypertensive males than in females. Studies from different populations have demonstrated a high percentage of TOD among hypertensive subjects [13-19]. Studies from the U.S.A. [13], Africa [14-17] and Europe [18,19] have showed high prevalence of TOD in hypertensive subjects. In addition, in African hypertensive subjects, males had higher odds of developing TOD compared to females [14]. It is well established that presense, even subclinical, of TOD is associated with increased cardiovascular mortality [4-9]. In addition, hypertensive subjects with LVH [4], increased IMT [5] and microalbuminuria [6-9] show increased cardiovascular risk. Therefore, presence of TOD has an important role in the treatment of hypertensive subjects and the prevention of cardiovascular disease [2,3]. Knowledge of prevalence of TOD in primary care might help to early detection and intensive treatment of subjects at high cardiovascular risk.

It is known that TOD already exists in newly diagnosed hypertensive subjects [20]. A recent study in our country showed that subjects with white coat and masked hypertension had TOD in terms of LVH and increased carotid IMT [21]. It is noteworthy, that even at early stages of hypertension TOD is present resulting at increased cardiovascular risk among hypertensive subjects [20,21]. Confirming previous studies, we showed that LVH was the most prevalent TOD in hypertensive subjects [13,15-17] followed by renal impairment [13,19].

Logistic regression analysis showed associations between TOD and presence of MS, dyslipidemia, DM and CAD. In hypertensive subjects MS, as a clustering of cardiovascular risk factors, amplifies TOD [22-24]. A recent study showed that the risk of LVH, carotid abnormalities and microalbuminuria increased by the presence of MS even after adjusting for several confounders [22]. Another study found a strong association between MS and TOD by showing that a clustering of two or three markers of TOD is the prevalent cardiovascular phenotype in MS hypertensive subjects [24]. A study in this elderly non-diabetic hypertensive subjects showed that the presence of MS was independently related to a greater prevalence of TOD and established cardiovascular disease [24].

In addition, DM and CAD per se have been associated with TOD in hypertensive subjects [16,19]. TOD tends to be more prevalent in hypertensive diabetic patients than in non-diabetics [25], as is the case in subjects with MS [26]. Another study found a relationship between TOD and established cardiovascular disease [19]. In fact, the prevalence of established cardiovascular disease was twice as great in hypertensive subjects with LVH and renal impairment [19]. Finally, despite previous findings, in the present study we failed to demonstrate any association between TOD and blood pressure levels [14,15].

## Limitations

The present study has its limitations. In the present study only subjects with a known history of arterial hypertension where included and therefore a selection bias could not be avoided. In addition, information regarding the duration of antihypertensive treatment and diabetes are missing. Another limitation is that measurements were obtained by biochemical laboratories at different regions and not a central one.

## Conclusion

In conclusion, a significant proportion of hypertensive subjects in primary care had documented associated TOD, with LVH being the most prevalent TOD. The above finding emphasizes the important role of the primary care clinicians to the early detection, treatment and control of high blood pressure that might help to reducing overall cardiovascular risk.

## Competing interests

The authors declare that they have no competing interests.

## Authors' contributions

ES, CP, AG, OA, HK and MS participated in the collection of the data. AP, ES, AS and CT participated in the design of the study and performed the statistical analysis and drafted the manuscript. All authors read and approved the final manuscript.

## Pre-publication history

The pre-publication history for this paper can be accessed here:

http://www.biomedcentral.com/1471-2296/12/75/prepub
